# Comparative efficacy of intra-articular polynucleotide injection combined with rehabilitation therapy in knee osteoarthritis with and without diabetes mellitus and construction of a nomogram: a propensity score-matched cohort study

**DOI:** 10.3389/fmed.2026.1906042

**Published:** 2026-09-25

**Authors:** Yi Wang, Li Chen, Yan Yan, Yishu Wen, Junjie Li, Wenming Bie, Kejie Shu

**Affiliations:** 1 Department of Orthopaedic Surgery, Chongqing Jiangbei Hospital of Traditional Chinese Medicine, Chongqing, China; 2 School of Orthopedics and Traumatology, Chongqing College of Chinese Medicine, Chongqing, China; 3 Orthopedics and Traumatology of Traditional Chinese Medicine Laboratory, Chongqing College of Chinese Medicine, Chongqing, China

**Keywords:** knee osteoarthritis, nomogram, polynucleotide, propensity score matching, type 2 diabetes mellitus

## Abstract

**Objective:**

To compare the longitudinal clinical efficacy of intra-articular polynucleotide (PN) injection combined with prescribed rehabilitation therapy in patients with knee osteoarthritis (KOA) with and without type 2 diabetes mellitus (T2DM), to investigate the influence of glycemic control on treatment outcomes, and to construct a predictive model.

**Methods:**

A total of 458 patients with KOA who received PN combined with rehabilitation therapy were retrospectively enrolled. After 1:2 propensity score matching (PSM), 270 patients were included (90 in the diabetes mellitus [DM] group and 180 in the non-DM group). Generalized estimating equations (GEE) were used to compare the longitudinal trajectories of Visual Analog Scale (VAS) and Western Ontario and McMaster Universities Osteoarthritis Index (WOMAC) scores between the two groups. Stratified analyses were further performed within the DM subgroup according to baseline glycated hemoglobin (HbA1c) levels. Least absolute shrinkage and selection operator (LASSO) regression combined with multivariable Logistic regression was used to construct a nomogram predicting the probability of achieving Patient Acceptable Symptom State (PASS) at 6 months.

**Results:**

After matching, no significant differences were observed between the two groups in the improvement trajectories of VAS and WOMAC scores (group effect, *P* > 0.05). Within the DM subgroup, the PASS achievement rate was significantly higher in patients with HbA1c < 7.0% than in those with HbA1c ≥ 7.0% (75.00% vs. 52.38%, *P* = 0.0248). The nomogram identified rehabilitation adherence (odds ratio [OR] = 2.95, 95% CI: 1.62–5.40), whereas uncontrolled DM (HbA1c ≥ 7.0%; OR = 0.40, 95% CI: 0.22–0.78), KL grade IV (OR = 0.35, 95% CI: 0.18–0.70), and BMI ≥ 28 kg/m^2^ (OR = 0.58, 95% CI: 0.35–0.96) were inversely associated with PASS achievement. The nomogram yielded a C-index of 0.82.

**Conclusion:**

Among patients with KOA receiving intra-articular PN injection together with a prescribed rehabilitation program, those with T2DM showed 6-month clinical outcome trajectories comparable to those without DM. Good glycemic control and favorable rehabilitation adherence are key factors for optimizing therapeutic outcomes.

## 1 Introduction

Knee osteoarthritis (KOA) is the most common degenerative joint disorder among middle-aged and older adults and is also one of the major causes of chronic pain and physical dysfunction (1). In recent years, with the deepening understanding of the pathological mechanisms of osteoarthritis, it has been gradually recognized that KOA is not merely a disease of “mechanical wear and tear”; rather, its onset and progression are also profoundly influenced by systemic metabolic factors. On this basis, the disease phenotype termed “metabolic osteoarthritis” has been proposed (2). Among the various metabolic risk factors, type 2 diabetes mellitus (T2DM) has been shown to be particularly closely associated with KOA. Epidemiological studies have demonstrated that the prevalence of KOA is significantly higher in patients with T2DM than in non-diabetic populations, and that joint degeneration tends to progress more rapidly in these patients (3, 4). Several potential mechanisms have been implicated. Long-term hyperglycemia promotes the accumulation of advanced glycation end-products (AGEs) in the cartilage matrix, resulting in stiffening of the collagen fiber network through non-enzymatic cross-linking and, consequently, impairment of the biomechanical buffering capacity of cartilage (5, 6). In addition, AGEs can bind to their specific receptor, receptor for AGEs (RAGE), thereby activating inflammatory signaling pathways such as nuclear factor kappa B (NF-κB), driving the sustained release of matrix metalloproteinases and pro-inflammatory mediators, and accelerating cartilage degradation (7). Furthermore, systemic insulin resistance further weakens the anti-inflammatory homeostatic capacity of synovial tissue (8). Therefore, patients with KOA complicated by T2DM generally bear a greater disease burden and experience poorer therapeutic outcomes.

In the vast majority of patients with KOA, particularly those classified as Kellgren–Lawrence (KL) grades I–III, conservative treatment based on a strategy of “basic treatment + pharmacological treatment” is primarily adopted, and intra-articular injection represents an important component of stepwise KOA management (9). However, existing injection-based approaches exhibit clear limitations in patients with T2DM. Although intra-articular corticosteroid injection can rapidly relieve inflammatory pain, it may induce marked fluctuations in blood glucose, particularly in patients with elevated baseline glycated hemoglobin (HbA1c), in whom the risks of glycemic decompensation and long-term infection are both significantly increased (10). Hyaluronic acid (HA) viscosupplementation can improve synovial fluid function from a rheological perspective; however, its biological half-life is relatively short, and it lacks the ability to directly intervene in metabolic inflammation driven by the AGEs–RAGE axis (11).

Polynucleotide (PN), a high-molecular-weight polymer derived from natural deoxyribonucleic acid (DNA), has attracted increasing attention in recent years as an intra-articular therapeutic agent for KOA. Clinical studies have shown that PN injection is comparable to, or even superior to, HA in reducing pain and improving joint function (12). Its mechanism of action appears to involve both physical and biological properties. On the one hand, by virtue of its high molecular weight and strong hydrophilicity, PN forms a viscoelastic gel within the joint cavity, thereby providing sustained hydrodynamic lubrication (11). On the other hand, PN has been shown to act as a selective agonist of the adenosine A2A receptor and to suppress NF-κB-mediated inflammatory responses through upregulation of the cyclic adenosine monophosphate (cAMP)/protein kinase A (PKA)/cAMP response element-binding protein (CREB) signaling pathway, promote macrophage polarization toward an anti-inflammatory phenotype, and stimulate chondrocytes to synthesize collagen and proteoglycans (13–15). This exogenous anti-inflammatory and pro-reparative mechanism, which is independent of autologous blood quality, theoretically renders PN a promising therapeutic option for patients with KOA complicated by T2DM.

Meanwhile, prescribed rehabilitation therapy based on the correction of lower-limb biomechanics constitutes the cornerstone of conservative treatment for KOA. Systematic quadriceps strengthening and proprioceptive training have been shown to effectively improve the dynamic stability of the knee joint and reduce abnormal mechanical loading across the articular surface (16). In clinical practice, injection therapy and rehabilitation training are frequently implemented as components of a combined conservative management strategy for KOA (17). However, real-world evidence remains limited regarding whether patients with T2DM can achieve clinical outcomes comparable to those of patients without DM when treated with this combined regimen. In addition, under the complex clinical background in which multiple factors intersect, the baseline characteristics capable of independently predicting clinical response after treatment remain to be systematically elucidated.

Accordingly, the present retrospective propensity score-matched cohort study was designed to compare the longitudinal clinical response trajectories of patients with KOA with and without T2DM who received the same combined regimen of intra-articular PN injection and prescribed rehabilitation therapy. The study did not aim to determine the incremental efficacy of rehabilitation beyond PN injection alone.

## 2 Materials and methods

### 2.1 Study design

This was a single-center retrospective observational cohort study based on the electronic medical records of patients with knee osteoarthritis who received at least one intra-articular polynucleotide injection at Chongqing Jiangbei Hospital of Traditional Chinese Medicine between January 2023 and August 2025. The last included patient completed the prescribed injection course by the end of August 2025. Follow-up data collection was completed, and the database was locked in March 2026. The study was designed to compare longitudinal clinical outcomes between patients with and without diabetes mellitus who received the same combined management strategy consisting of intra-articular polynucleotide injection and prescribed rehabilitation therapy. The study protocol was approved by the Ethics Committee of Chongqing Jiangbei Hospital of Traditional Chinese Medicine (approval No.JZY-IRB-202527), and all procedures were conducted in strict accordance with the Declaration of Helsinki and relevant guidelines. Given the retrospective design of the study, the requirement for informed consent was waived.

### 2.2 Study population

The inclusion criteria were as follows: (1) age ≥ 50 years; (2) fulfillment of the clinical diagnostic criteria for KOA established by the ACR; (3) radiographic KL grade II–IV; (4) completion of a full course of intra-articular PN injection (once weekly for 3–5 consecutive weeks); (5) receipt of prescribed non-surgical rehabilitation guidance after injection, including lower-limb biomechanical correction programs such as quadriceps isometric contraction training and proprioceptive training; and (6) availability of complete follow-up data at baseline, 3 months after the final PN injection, and 6 months after intervention.

The exclusion criteria were as follows: (1) concomitant inflammatory arthropathies of the knee, including septic arthritis, rheumatoid arthritis, and gouty arthritis; (2) receipt of knee surgery or other intra-articular injection therapies during the study period, such as HA, corticosteroids, or platelet-rich plasma; (3) concomitant severe systemic diseases that might affect overall functional status, including severe cardiovascular or cerebrovascular disease, malignant tumors, and severe hepatic or renal insufficiency; and (4) incomplete follow-up data or loss to follow-up. Follow-up was considered incomplete if the scheduled 3-month assessment, the scheduled 6-month assessment, or both assessments were unavailable because of missing records or loss to follow-up. Patients missing only the 3-month assessment, only the 6-month assessment, or both follow-up assessments were excluded from the final complete-case cohort.

Because the predefined target population comprised patients treated under a combined management strategy involving PN injection and a prescribed rehabilitation program, patients who received PN injection without being referred to the rehabilitation program were not included in the analytic cohort.

Group definitions were determined according to the presence or absence of T2DM. The DM group was defined as patients with a prior confirmed diagnosis of T2DM who received standardized glucose-lowering treatment during the study period. The non-DM group was defined as patients without diabetes or other severe metabolic disorders. The diagnosis of DM was based on World Health Organization (WHO) criteria or on a clearly documented diagnosis in the prior medical record.

### 2.3 Intervention protocol

#### 2.3.1 Injection phase

All patients received intra-articular PN injection once weekly for 3–5 consecutive weeks. The exact number of injections was determined according to clinical condition and physician judgment. All injection procedures were performed under standard aseptic conditions by orthopedic or rehabilitation physicians with extensive experience in intra-articular injection.

#### 2.3.2 Treatment and follow-up timeline

Baseline assessments were performed before the first intra-articular PN injection. The date of the final PN injection was defined as the index date for follow-up. The prescribed 12-week rehabilitation program commenced after completion of the injection series. The 3-month and 6-month follow-up assessments were performed 3 and 6 months after the final PN injection, respectively. Therefore, the 3-month assessment approximately corresponded to completion of the prescribed 12-week rehabilitation period.

#### 2.3.3 Rehabilitation phase

After completion of the injection course, all patients were referred to and received an individualized prescription for a 12-week rehabilitation program. The prescribed program comprised four principal components: (1) quadriceps isometric contraction and progressive resistance training; (2) knee proprioceptive training; (3) lower-limb biomechanical correction training; and (4) activity modification and joint-protection education. Individualized prescriptions were developed by rehabilitation therapists, and regular follow-up guidance was provided. Because the extent to which the prescribed components were actually implemented could vary among patients, prescription and documented implementation were evaluated separately.

#### 2.3.4 Documentation of rehabilitation implementation

To distinguish the prescribed rehabilitation program from its actual implementation in clinical care, the available rehabilitation treatment records and follow-up documentation in the electronic medical record were retrospectively reviewed for each patient in the matched cohort. Each of the four rehabilitation components was separately coded as “documented as performed” or “not documented as performed.” A component was classified as documented as performed only when its implementation was explicitly recorded in the rehabilitation or follow-up documentation; prescription alone was not considered evidence of implementation. The proportions of patients with documented implementation of each component and of all four components were calculated for the overall matched cohort and separately for the DM and non-DM groups.

#### 2.3.5 Rehabilitation adherence assessment

Rehabilitation adherence was assessed over the 12-week prescribed rehabilitation period using rehabilitation attendance records and outpatient revisit logs. A total of 12 rehabilitation visits were scheduled for each patient during this period. Good rehabilitation adherence was predefined as attendance at 9 or more of the 12 scheduled visits, corresponding to an attendance rate of at least 75%. Patients attending 8 or fewer scheduled visits were classified as having poor rehabilitation adherence. Component-level implementation of the four prescribed rehabilitation activities was evaluated separately and was not used to determine the binary adherence classification. Rehabilitation adherence was entered into the predictive analysis as a binary variable, with good adherence coded as 1 and poor adherence coded as 0.

### 2.4 Outcome measures

The primary outcome measures were the Visual Analog Scale pain score and the total Western Ontario and McMaster Universities Osteoarthritis Index score. Baseline assessments were conducted before the first PN injection. Follow-up assessments were conducted 3 and 6 months after the final injection of the PN series. The 3-month assessment approximately coincided with completion of the prescribed 12-week rehabilitation period.

Advanced clinical response indicators included: (1) the proportion of patients achieving the minimal clinically important difference (MCID); (2) the clinical response rate according to the Outcome Measures in Rheumatology–Osteoarthritis Research Society International (OMERACT–OARSI) responder criteria; and (3) the proportion of patients achieving the Patient Acceptable Symptom State (PASS) at 6 months after the final PN injection, defined as a Visual Analog Scale score ≤ 3 (18).

PASS was selected as the binary outcome for predictive model development because the objective of the nomogram was to estimate the probability that an individual patient would reach a clinically acceptable absolute pain state at the fixed 6-month endpoint. In contrast, MCID is a change-based measure that reflects improvement relative to baseline and may be achieved despite clinically relevant residual pain, whereas the OMERACT–OARSI responder definition is a composite measure that may be fulfilled through different combinations of changes across outcome domains. PASS therefore provided a single, clinically interpretable endpoint aligned with the intended application of the prediction model. MCID achievement and OMERACT–OARSI response were retained as complementary secondary indicators of treatment response.

For secondary and safety outcomes, baseline HbA1c was measured before the first PN injection, and the follow-up HbA1c value was obtained 6 months after the final PN injection.

### 2.5 Sample size and data source

During the study period, 650 patients with knee osteoarthritis who received at least one intra-articular polynucleotide injection were identified from the hospital electronic medical record system and assessed for eligibility. Before assessment of follow-up completeness, 147 patients were excluded: 25 did not complete the prescribed polynucleotide injection course, 46 were not referred to or did not enter the prescribed rehabilitation program, and 76 met other clinical exclusion criteria, including concomitant inflammatory arthropathy, receipt of knee surgery or other intra-articular injection therapies, or severe systemic disease.

The remaining 503 patients otherwise met the eligibility criteria. Among them, 45 patients (8.95%) were excluded because of incomplete follow-up data: 12 were missing only the 3-month assessment, 21 were missing only the 6-month assessment, and 12 were missing both assessments. Consequently, 458 patients, representing 91.05% of the otherwise eligible population, were included in the final unmatched cohort, comprising 105 patients with diabetes mellitus and 353 patients without diabetes mellitus. The detailed patient selection process is presented in Figure 1.

**Figure 1:**
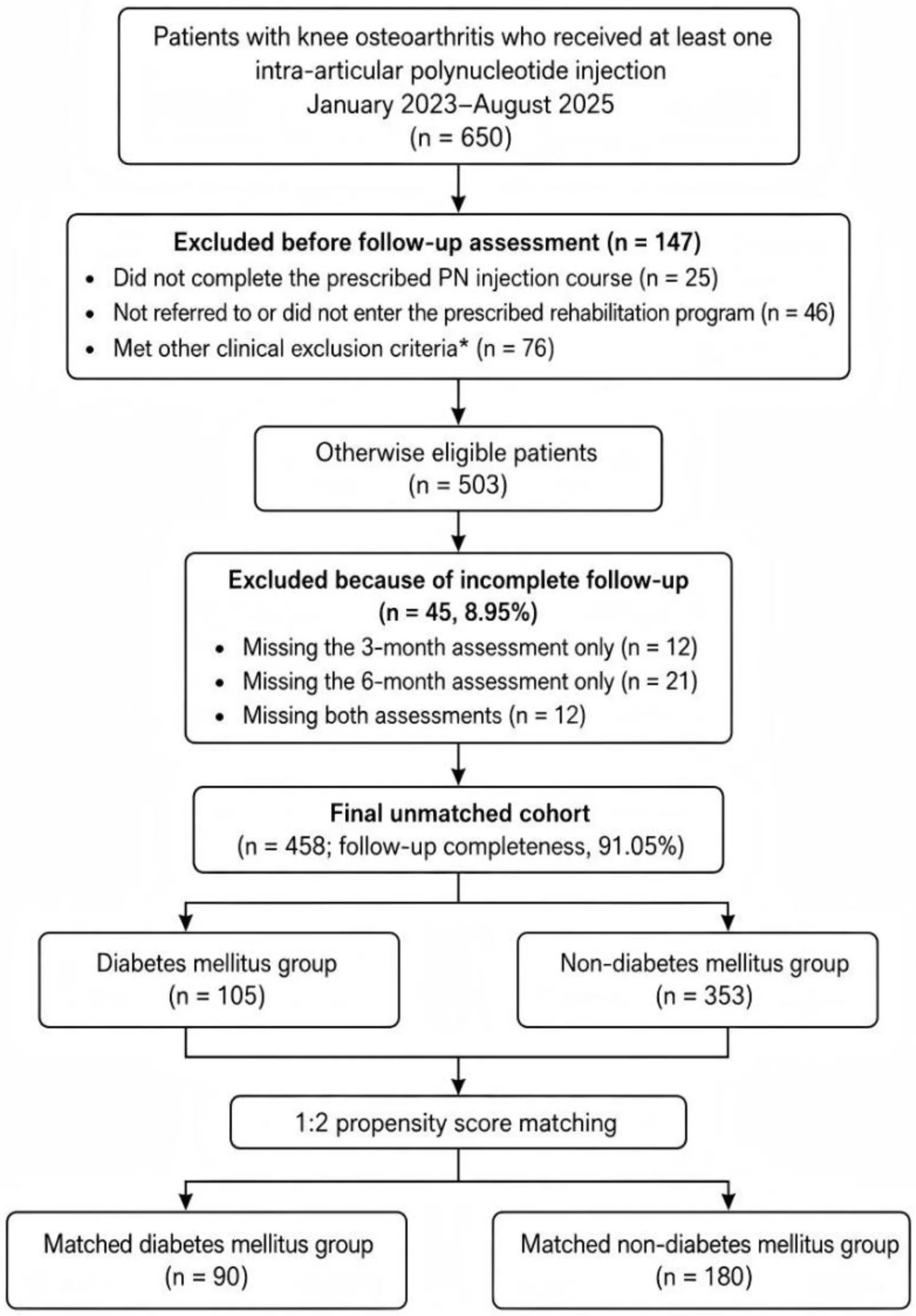
Flow diagram of patient identification, eligibility assessment, follow-up completeness, and propensity score matching. A total of 650 patients with knee osteoarthritis who received at least one intra-articular polynucleotide injection between January 2023 and August 2025 were screened. After application of the clinical eligibility criteria and assessment of follow-up completeness, 458 patients were included in the unmatched cohort, of whom 270 were retained after 1:2 propensity score matching. PN, polynucleotide; PSM, propensity score matching.

Demographic characteristics, radiographic data, laboratory findings, clinical scores, treatment records, rehabilitation records, and follow-up outcomes were extracted from the hospital electronic medical record system.

### 2.6 Statistical methods

#### 2.6.1 Propensity score matching

To control the inherent selection bias and confounding factors in retrospective research, PSM was used to balance baseline characteristics between the two groups. With the presence of DM as the dependent variable, seven covariates were entered into a Logistic regression model to calculate the propensity score for each patient, including age, sex, BMI, KL grade, baseline VAS score, baseline WOMAC score, and number of intra-articular PN injections. A 1:2 nearest-neighbor matching method with a caliper width of 0.02 was then applied to match the DM and non-DM groups. Matching quality was evaluated using the standardized mean difference (SMD), and an SMD < 0.10 was considered indicative of good balance for each covariate.

#### 2.6.2 Evaluation of longitudinal clinical outcomes

For repeated continuous measurements obtained at baseline, 3 months after the final PN injection, and 6 months after intervention (VAS and WOMAC scores), GEE was used for longitudinal analysis, with an exchangeable correlation structure specified as the working correlation matrix. Time effect, group effect (DM vs. non-DM), and the time × group interaction term were included in the model to systematically assess whether the clinical improvement trajectories differed between the two groups. For discrete clinical response indicators (MCID achievement rate, OMERACT-OARSI response rate, and PASS achievement rate), the chi-square test or Fisher's exact test was used for between-group comparison. Documented implementation rates for the individual rehabilitation components were summarized as numbers and percentages and compared between the DM and non-DM groups using the chi-square test with Yates continuity correction.

#### 2.6.3 Metabolic subgroup analysis

To explore the potential influence of glycemic control on the efficacy of PN combined with rehabilitation therapy, the DM group was stratified according to baseline HbA1c level into a subgroup with HbA1c < 7.0% (good glycemic control) and a subgroup with HbA1c ≥ 7.0% (poor glycemic control). Continuous variables were compared between the two HbA1c subgroups using the independent-samples t-test or Mann–Whitney U test, as appropriate. The proportions of patients achieving the minimal clinically important difference (MCID), meeting the Outcome Measures in Rheumatology–Osteoarthritis Research Society International (OMERACT–OARSI) responder criteria, and achieving the Patient Acceptable Symptom State (PASS) at 6 months after the final PN injection were compared using Pearson's chi-square test when all expected cell counts were ≥5; otherwise, Fisher's exact test was used. Changes in HbA1c before and after intervention within the DM group were also evaluated to examine the endocrine safety of intra-articular PN injection.

#### 2.6.4 Feature selection based on LASSO regression and construction of a multivariable predictive model

To identify factors associated with achieving PASS at 6 months, a two-stage modeling strategy was adopted. In the first stage, 15 candidate clinical variables from the fully matched cohort (*N* = 270) were entered into a least absolute shrinkage and selection operator (LASSO) regression model. These variables included age, sex, BMI ≥ 28 kg/m^2^, symptom duration, Kellgren–Lawrence grade IV, bilateral knee involvement, type 2 diabetes mellitus status, uncontrolled diabetes mellitus defined as glycated hemoglobin ≥ 7.0%, hypertension, hyperlipidemia, baseline Visual Analog Scale score, baseline Western Ontario and McMaster Universities Osteoarthritis Index score, number of intra-articular polynucleotide injections, rehabilitation adherence, and concomitant oral nonsteroidal anti-inflammatory drug use. The number of intra-articular polynucleotide injections was modeled as a continuous variable ranging from 3 to 5. Ten-fold cross-validation was used to select the penalty parameter according to the 1-standard-error rule. Variables with non-zero coefficients at λ1se were retained for subsequent analysis. At λ1se = 0.0385, the coefficient for the number of intra-articular polynucleotide injections was shrunk to zero; therefore, it was not entered into the subsequent multivariable logistic regression model or included in the final nomogram.

In the second stage, the core variables selected by LASSO were entered into a multivariable Logistic regression model, and the OR and corresponding 95%CI were calculated for each independent predictor. Based on the Logistic regression results, a clinical nomogram was constructed to predict the probability of achieving PASS at 6 months after intervention. Model discrimination was evaluated using the C-index, which is equivalent to the AUC. Calibration was assessed by calibration curves generated through 1,000 bootstrap resamples and by the Hosmer–Lemeshow goodness-of-fit test. Clinical utility was validated by DCA through net benefit across different threshold probabilities.

#### 2.6.5 Statistical software and significance threshold

All statistical analyses were performed using R software (version 4.2.2) and SPSS software (version 22.1). The main R packages included MatchIt (for PSM), geepack (for GEE), glmnet (for LASSO regression), rms (for nomogram and calibration curve construction), and rmda (for DCA). A two-sided P value < 0.05 was considered statistically significant.

## 3 Results

### 3.1 Participant screening, characteristics, and propensity score matching

Between January 2023 and August 2025, 650 patients with knee osteoarthritis who received at least one intra-articular polynucleotide injection were screened for eligibility. Of these, 25 patients were excluded because they did not complete the prescribed injection course, 46 because they were not referred to or did not enter the prescribed rehabilitation program, and 76 because they met other clinical exclusion criteria. The remaining 503 patients otherwise met the eligibility criteria. Among them, 45 patients (8.95%) were excluded because of incomplete follow-up data, including 12 who were missing only the 3-month assessment, 21 who were missing only the 6-month assessment, and 12 who were missing both assessments. Therefore, 458 patients were included in the final unmatched cohort, representing a follow-up completeness rate of 91.05% among otherwise eligible patients (Figure 1). The cohort included 105 patients with diabetes mellitus (22.93%) and 353 patients without diabetes mellitus (77.07%).

However, before matching, significant between-group differences were present in several key baseline characteristics. The mean age of patients in the DM group was significantly higher than that of patients in the non-DM group (67.25 ± 5.40 years vs. 64.10 ± 6.20 years, *P* < 0.0001), and BMI was also significantly greater in the DM group (27.85 ± 3.45 kg/m^2^ vs. 25.10 ± 3.10 kg/m^2^, *P* < 0.0001). With respect to baseline pain and functional indices, the DM group also exhibited higher VAS scores (6.85 ± 1.15 vs. 6.40 ± 1.25, *P* = 0.0012) and higher total WOMAC scores (58.45 ± 8.75 vs. 55.20 ± 9.45, *P* = 0.0018), suggesting that patients with DM-associated KOA bore a heavier baseline disease burden. Regarding radiographic grading, although the overall distribution of KL grade did not differ significantly between the two groups (*P* = 0.1245), a higher proportion of KL grade IV cases (severe degeneration) was observed in the DM group than in the non-DM group (19.05% vs. 12.75%).

To effectively control the potential confounding bias introduced by these baseline differences, 1:2 nearest-neighbor PSM was performed using age, sex, BMI, KL grade, baseline VAS, baseline WOMAC, and number of PN injections as covariates, with a caliper width of 0.02. After matching, 270 patients were ultimately included, comprising 90 patients in the DM group and 180 in the non-DM group. After matching, no statistically significant differences were observed in any of the matched baseline covariates between the two groups. In particular, the distribution of Kellgren–Lawrence grades was comparable between the DM and non-DM groups (grade II: 35.56% vs. 36.67%; grade III: 46.67% vs. 46.11%; grade IV: 17.78% vs. 17.22%; overall *P* = 0.9654; SMD = 0.035). The standardized mean differences for all matched covariates ranged from 0.018 to 0.048, all below the prespecified threshold of 0.10, indicating adequate post-matching balance. In addition, the SMDs for all covariates were controlled within 0.05 (range: 0.018–0.048), far below the commonly accepted balance threshold of 0.10, confirming that excellent baseline balance had been achieved in the matched cohort (Table 1).

**Table 1:** Baseline demographic and clinical characteristics before and after 1:2 propensity score matching.

Variables	Before PSM			After PSM (1:2, *N* = 270)			
	DM (*n* = 105)	Non-DM (*n* = 353)	*P*-value	DM (*n* = 90)	Non-DM (*n* = 180)	*P*-value	SMD
Age (years, mean ± SD)	67.25 ± 5.40	64.10 ± 6.20	<0.0001	65.80 ± 5.50	65.70 ± 5.35	0.8845	0.018
Female, *n* (%)	65 (61.90)	201 (56.94)	0.3621	54 (60.00)	106 (58.89)	0.8521	0.022
BMI (kg/m^2^, mean ± SD)	27.85 ± 3.45	25.10 ± 3.10	<0.0001	26.55 ± 3.15	26.40 ± 3.05	0.7056	0.048
KL grade, *n* (%)			0.1245			0.9654	0.035
Grade II	35 (33.33)	148 (41.93)		32 (35.56)	66 (36.67)		
Grade III	50 (47.62)	160 (45.33)		42 (46.67)	83 (46.11)		
Grade IV	20 (19.05)	45 (12.75)		16 (17.78)	31 (17.22)		
Baseline VAS	6.85 ± 1.15	6.40 ± 1.25	0.0012	6.60 ± 1.10	6.58 ± 1.12	0.8872	0.018
Baseline WOMAC	58.45 ± 8.75	55.20 ± 9.45	0.0018	56.50 ± 8.20	56.25 ± 8.35	0.8123	0.030
Intra-articular PN (*n*)	4.15 ± 0.80	4.05 ± 0.85	0.2815	4.10 ± 0.80	4.12 ± 0.78	0.8415	0.025

PSM, propensity score matching; SMD, standardized mean difference; DM, diabetes mellitus; SD, standard deviation; BMI, body mass index; KL, Kellgren–Lawrence; VAS, visual analog scale; WOMAC, Western Ontario and McMaster Universities Osteoarthritis Index; PN, polynucleotide.

### 3.2 Documented implementation of the prescribed rehabilitation program

Among the 270 patients in the matched cohort, quadriceps isometric and progressive resistance training was documented as performed in 230 patients (85.2%), proprioceptive training in 208 patients (77.0%), lower-limb biomechanical correction training in 194 patients (71.9%), and activity modification and joint-protection education in 219 patients (81.1%). Implementation of all four prescribed components was documented in 167 patients (61.9%).

The documented implementation rates were comparable between the DM and non-DM groups for quadriceps isometric and progressive resistance training (83.3% vs. 86.1%, *P* = 0.672), proprioceptive training (74.4% vs. 78.3%, *P* = 0.574), lower-limb biomechanical correction training (68.9% vs. 73.3%, *P* = 0.534), activity modification and joint-protection education (78.9% vs. 82.2%, *P* = 0.621), and all four components combined (60.0% vs. 62.8%, *P* = 0.757). These findings indicated no significant between-group imbalance in the documented implementation of the prescribed rehabilitation components (Table 2). In the matched cohort, 162 patients (60.0%) were classified as having good rehabilitation adherence, defined as attendance at 9 or more of the 12 scheduled rehabilitation visits, whereas 108 patients (40.0%) were classified as having poor adherence. Good adherence was observed in 55 of 90 patients (61.1%) in the DM group and 107 of 180 patients (59.4%) in the non-DM group, with no significant between-group difference (*P* = 0.895).

**Table 2:** Documented implementation of the prescribed rehabilitation components in the matched cohort.

Rehabilitation component	Overall cohort, *n* (%) (*N* = 270)	DM group, *n* (%) (*n* = 90)	Non-DM group, *n* (%) (*n* = 180)	*P* value
Quadriceps isometric and progressive resistance training	230 (85.2)	75 (83.3)	155 (86.1)	0.672
Proprioceptive training	208 (77.0)	67 (74.4)	141 (78.3)	0.574
Lower-limb biomechanical correction training	194 (71.9)	62 (68.9)	132 (73.3)	0.534
Activity modification and joint-protection education	219 (81.1)	71 (78.9)	148 (82.2)	0.621
All four components documented	167 (61.9)	54 (60.0)	113 (62.8)	0.757
Good rehabilitation adherence	162 (60.0)	55 (61.1)	107 (59.4)	0.895
Poor rehabilitation adherence	108 (40.0)	35 (38.9)	73 (40.6)	–

Good rehabilitation adherence was defined as attendance at ≥9 of the 12 scheduled rehabilitation visits during the 12-week rehabilitation period; attendance at ≤8 visits was classified as poor adherence. Component-level implementation of the four prescribed rehabilitation activities was assessed separately and was not used to define good or poor adherence. The *P* value was calculated using the chi-square test with Yates continuity correction.

DM, diabetes mellitus.

### 3.3 Comparison of longitudinal clinical responses

GEE based on an exchangeable correlation structure was used to analyze the repeated clinical measurements. The results showed that VAS and WOMAC scores in both groups exhibited significant time-dependent declines during follow-up (time effect, *P* < 0.0001). At both 3 months and 6 months after the final PN injection, no statistically significant between-group difference (DM vs. non-DM) was observed (group effect: VAS, *P* = 0.5842; WOMAC, *P* = 0.6215), and the time × group interaction was likewise non-significant (*P* > 0.05).

With respect to the discrete clinical response indicators at 6 months after the final PN injection, 61 of 90 patients (67.78%) in the DM group and 128 of 180 patients (71.11%) in the non-DM group achieved the minimal clinically important difference (*P* = 0.5732). The OMERACT–OARSI responder rates were 70.00% (63/90) and 73.33% (132/180), respectively (*P* = 0.5645). Similarly, PASS was achieved by 58 of 90 patients (64.44%) in the DM group and 122 of 180 patients (67.78%) in the non-DM group, with no statistically significant between-group difference (*P* = 0.5847). These findings indicated that patients with KOA with and without DM showed comparable clinical response rates during the 6 months following the final PN injection (Table 3; Figure 2).

**Table 3:** Longitudinal clinical outcomes and responder rates between DM and non-DM groups (*N* = 270).

Outcomes	DM group (*n* = 90)	Non-DM group (*n* = 180)	*P*-value (group diff)
VAS score			0.5842*
Baseline	6.60 ± 1.10	6.58 ± 1.12	0.8872
3 Months	3.45 ± 1.25	3.35 ± 1.18	0.5144
6 Months	2.85 ± 1.30	2.70 ± 1.25	0.3645
WOMAC score			0.6215*
Baseline	56.50 ± 8.20	56.25 ± 8.35	0.8123
3 Months	34.20 ± 9.15	33.10 ± 8.95	0.3421
6 Months	25.80 ± 9.40	24.50 ± 9.10	0.2745
Responder rates, *n* (%)			
Achieved MCID at 6M	61 (67.78)	128 (71.11)	0.5732
OMERACT-OARSI responder at 6M	63 (70.00)	132 (73.33)	0.5645
PASS (VAS ≤ 3) at 6M	58 (64.44)	122 (67.78)	0.5847

DM, diabetes mellitus; VAS, visual analog scale; WOMAC, Western Ontario and McMaster Universities Osteoarthritis Index; GEE, generalized estimating equations; MCID, minimal clinically important difference; OMERACT–OARSI, Outcome Measures in Rheumatology–Osteoarthritis Research Society International; PASS, Patient Acceptable Symptom State.

* *P*-value represents the main group effect derived from the GEE model with an exchangeable working correlation matrix.

**Figure 2:**
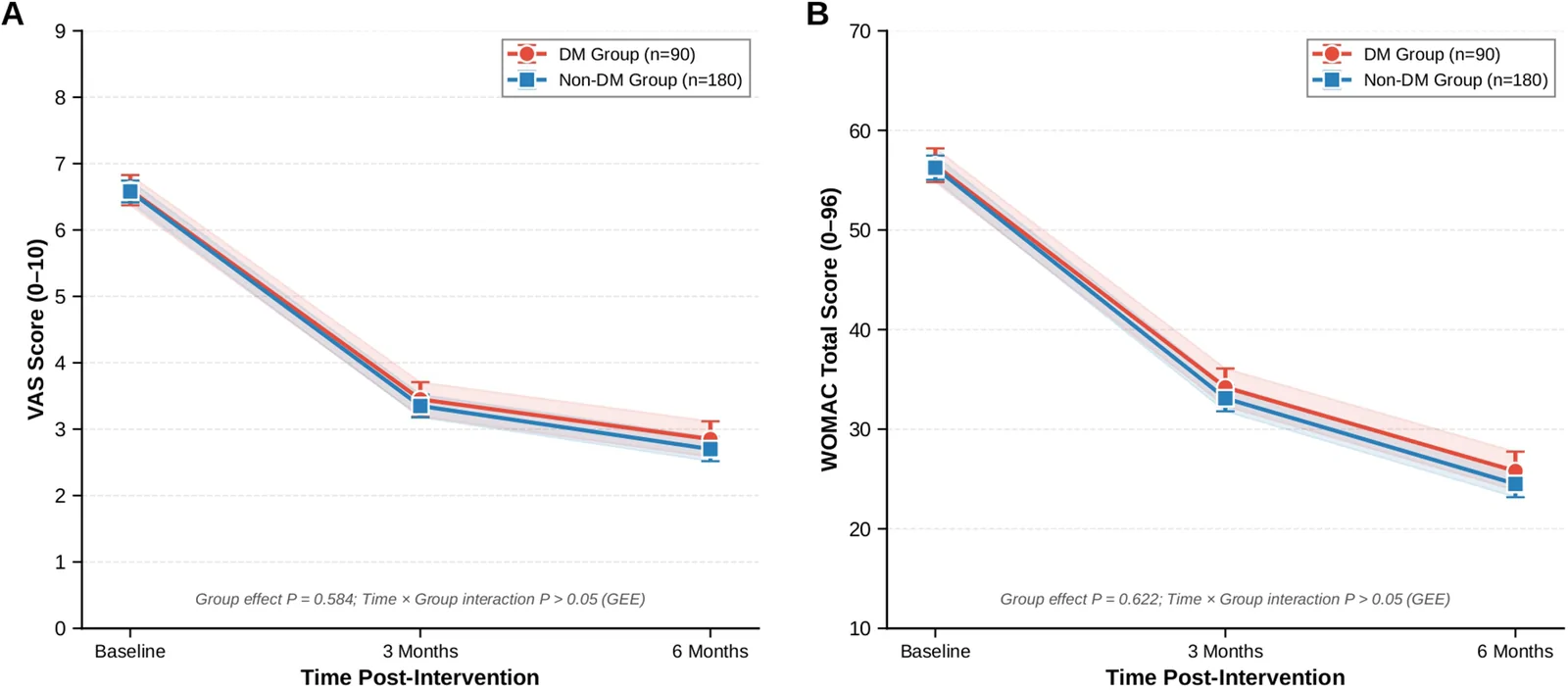
Longitudinal clinical improvements between DM and non-DM groups post-PSM (1:2). **(A)** Trajectories of mean Visual Analog Scale (VAS) scores from baseline to 6 months post-intervention. **(B)** Trajectories of mean WOMAC total scores. Error bars represent 95% Confidence Intervals (CI) to illustrate estimate precision. No significant group-by-time interaction or main group effects were observed (all *P* > 0.05 via Generalized Estimating Equations). DM, diabetes mellitus; VAS, visual analog scale; WOMAC, Western Ontario and McMaster Universities Osteoarthritis Index; CI, confidence interval; GEE, generalized estimating equations.

### 3.4 Metabolic status subgroup analysis

No evidence was observed in this cohort that intra-articular PN injection induced abnormal glycemic metabolism. HbA1c levels in the DM group remained stable before and after intervention (baseline: 6.95 ± 0.85% vs. 6 months: 6.90 ± 0.90%, *P* = 0.4215).

Stratified analysis within the DM group (*n* = 90) according to baseline HbA1c level revealed that different glycemic control states influenced the rate of early clinical symptom relief. As shown in Table 4, although no between-subgroup difference was observed in baseline disease severity, the WOMAC score at 3 months after the final PN injection was significantly lower in the HbA1c < 7.0% subgroup (*n* = 48) than in the HbA1c ≥ 7.0% subgroup (*n* = 42) (31.85 ± 8.20 vs. 36.85 ± 9.65, *P* = 0.0125). At 6 months after the final PN injection, the MCID achievement rate was significantly higher in the HbA1c < 7.0% subgroup than in the HbA1c ≥ 7.0% subgroup. MCID was achieved by 37 of 48 patients (77.08%) in the HbA1c < 7.0% subgroup and 24 of 42 patients (57.14%) in the HbA1c ≥ 7.0% subgroup (*P* = 0.0434). The HbA1c < 7.0% subgroup also showed higher OMERACT–OARSI responder and PASS achievement rates than the HbA1c ≥ 7.0% subgroup (79.17% vs. 59.52%, *P* = 0.0435; and 75.00% vs. 52.38%, *P* = 0.0248, respectively).

**Table 4:** Subgroup clinical responses within the DM cohort based on baseline HbA1c levels (*n* = 90).

Variables	HbA1c < 7.0% (*n* = 48)	HbA1c ≥ 7.0% (*n* = 42)	*P*-value
Baseline WOMAC	56.10 ± 8.05	56.95 ± 8.45	0.6214
WOMAC at 3 months	31.85 ± 8.20	36.85 ± 9.65	0.0125
WOMAC at 6 months	23.90 ± 8.45	27.95 ± 10.10	0.0412
WOMAC reduction (0–3M)	24.25 ± 6.15	20.10 ± 5.80	0.0015
Achieved MCID at 6 months after the final PN injection, *n* (%)	37 (77.08)	24 (57.14)	0.0434
OMERACT-OARSI responder at 6M, *n* (%)	38 (79.17)	25 (59.52)	0.0435
PASS (VAS ≤ 3) at 6M, *n* (%)	36 (75.00)	22 (52.38)	0.0248

DM, diabetes mellitus; HbA1c, glycated hemoglobin; WOMAC, Western Ontario and McMaster Universities Osteoarthritis Index; OMERACT–OARSI, Outcome Measures in Rheumatology–Osteoarthritis Research Society International; PASS, Patient Acceptable Symptom State; VAS, visual analog scale.

### 3.5 Multivariable predictive model of therapeutic efficacy

To identify the independent determinants of failure to achieve PASS at 6 months after the final PN injection, 15 candidate clinical features from the fully matched cohort (*N* = 270), covering demographic, imaging, metabolic, and rehabilitation adherence characteristics, were entered into a LASSO penalized regression model to avoid multicollinearity. Through 10-fold cross-validation (mean squared error, 1-standard-error path selection, λ₁ₛₑ = 0.0385), coefficients of non-essential redundant variables were shrunk to zero, and four core features with non-zero coefficients were ultimately extracted (Figure 3). The number of intra-articular PN injections was included among the 15 initial candidate variables, but its coefficient was shrunk to zero at λ1se. Consequently, the number of injections was not retained in the multivariable logistic regression model and was not incorporated into the final nomogram. The four retained variables were good rehabilitation adherence, uncontrolled DM, KL grade IV, and BMI ≥ 28 kg/m^2^.

**Figure 3:**
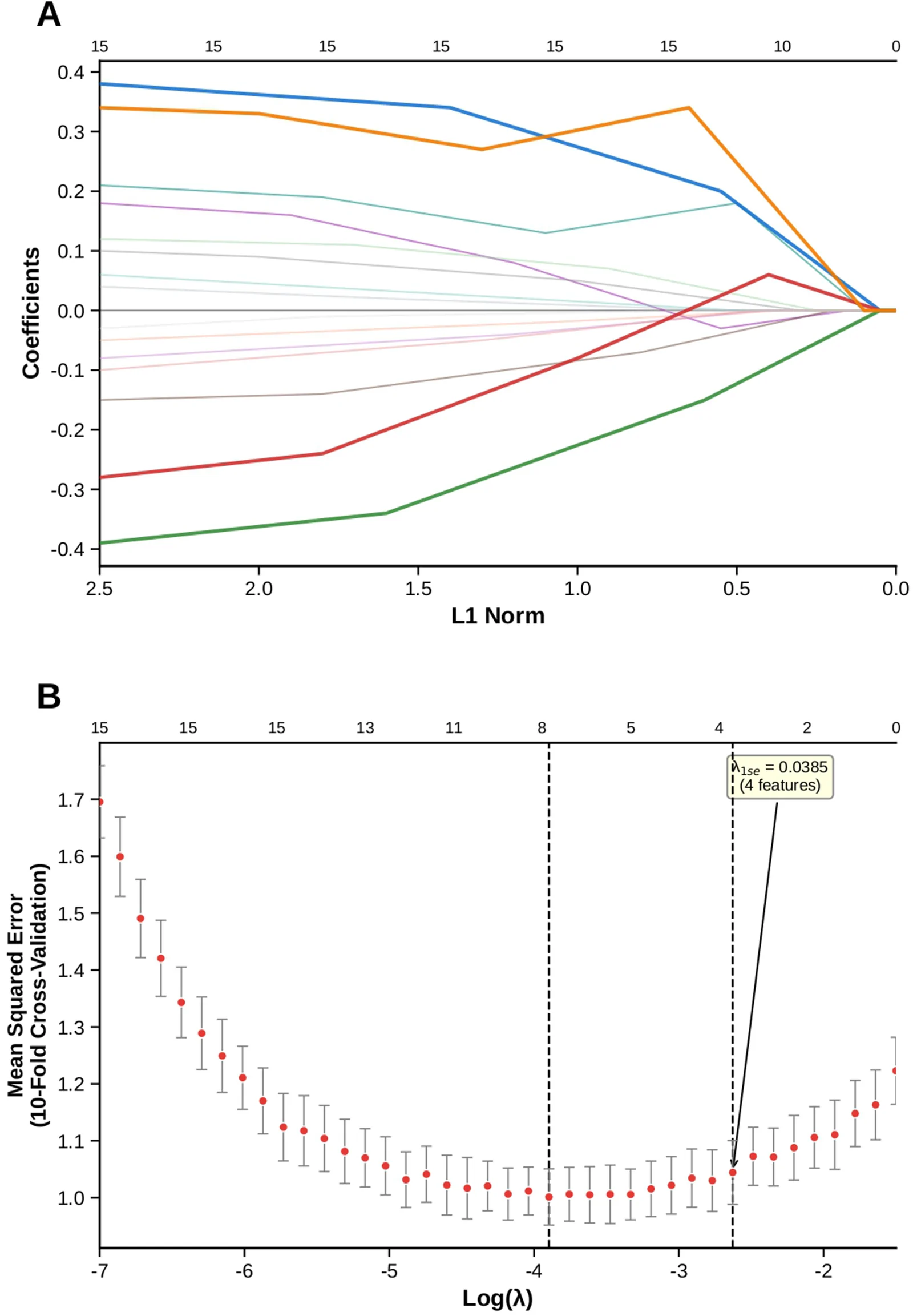
Feature dimensionality reduction using LASSO regression in the overall matched cohort (*N* = 270). **(A)** Coefficient shrinkage profiles across various log(λ) tuning parameters. **(B)** Ten-fold cross-validation mean squared error curve. The dashed vertical line on the right represents the optimal λ selected via the 1-standard-error rule (λ1se), which robustly identified four non-zero clinical features. The number of intra-articular PN injections was included among the 15 candidate variables but was not retained at λ1se because its coefficient was shrunk to zero. LASSO, least absolute shrinkage and selection operator; λ, penalty parameter; CV, cross-validation; 1-SE, one-standard-error.

Subsequent multivariable Logistic regression analysis (Table 5) confirmed that good adherence to prescribed rehabilitation was a strong independent protective factor for achieving PASS (OR = 2.95, 95% CI: 1.62–5.40, *P* = 0.0004). In contrast, uncontrolled DM (defined as DM with HbA1c ≥ 7.0%; OR = 0.40, 95% CI: 0.22–0.78, *P* = 0.0085), severe knee degeneration (KL grade IV; OR = 0.35, 95% CI: 0.18–0.70, *P* = 0.0031), and obesity (BMI ≥ 28 kg/m^2^; OR = 0.58, 95% CI: 0.35–0.96, *P* = 0.0345) were identified as significant independent risk factors impeding the attainment of satisfactory efficacy.

**Table 5:** Final multivariable logistic regression model for achieving PASS at 6 months after LASSO feature selection.

Core predictors (retained by LASSO)	Coefficient (*β*)	Standard error	Odds ratio (OR)	95% CI	*P*-value
*Protective factor*					
Good rehabilitation Adherence	1.0818	0.3065	2.95	1.62–5.40	0.0004
*Risk factors*					
Uncontrolled DM (HbA1c ≥ 7.0%)*	−0.9163	0.3235	0.40	0.22–0.78	0.0085
KL grade IV	−1.0498	0.3461	0.35	0.18–0.70	0.0031
Baseline BMI ≥ 28 kg/m^2^	−0.5447	0.2575	0.58	0.35–0.96	0.0345

LASSO, least absolute shrinkage and selection operator; PASS, Patient Acceptable Symptom State; OR, odds ratio; CI, confidence interval; DM, diabetes mellitus; HbA1c, glycated hemoglobin; KL, Kellgren–Lawrence; BMI, body mass index.

*Uncontrolled DM was defined as T2DM with baseline HbA1c ≥ 7.0%; the reference category comprised patients without DM and patients with T2DM with HbA1c < 7.0%.

To translate this multivariable predictive model into a tool convenient for clinical use, a clinical nomogram predicting the probability of achieving PASS at 6 months after the final PN injection was constructed on the basis of the Logistic regression results (Figure 4A). Overall model performance showed that the predictive model had a C-index (equivalent to the AUC) of 0.82 (95% CI: 0.76–0.88), indicating excellent discrimination. Based on commonly used interpretive criteria, a discrimination statistic between 0.80 and 0.90 is considered excellent (19, 20). To further validate model accuracy, a calibration curve generated by 1,000 bootstrap resamples was plotted (Figure 4B), and the predicted probabilities showed a high degree of agreement with the actually observed PASS rates (Hosmer–Lemeshow goodness-of-fit test, *P* = 0.6124). In addition, DCA (Figure 4C) confirmed that, over a wide range of threshold probabilities (approximately 10%–85%), the net benefit derived from applying this nomogram to guide clinical intervention decisions was significantly greater than that of the two extreme strategies of “treat all” or “treat none,” thereby demonstrating substantial practical value in real-world clinical settings.

**Figure 4:**
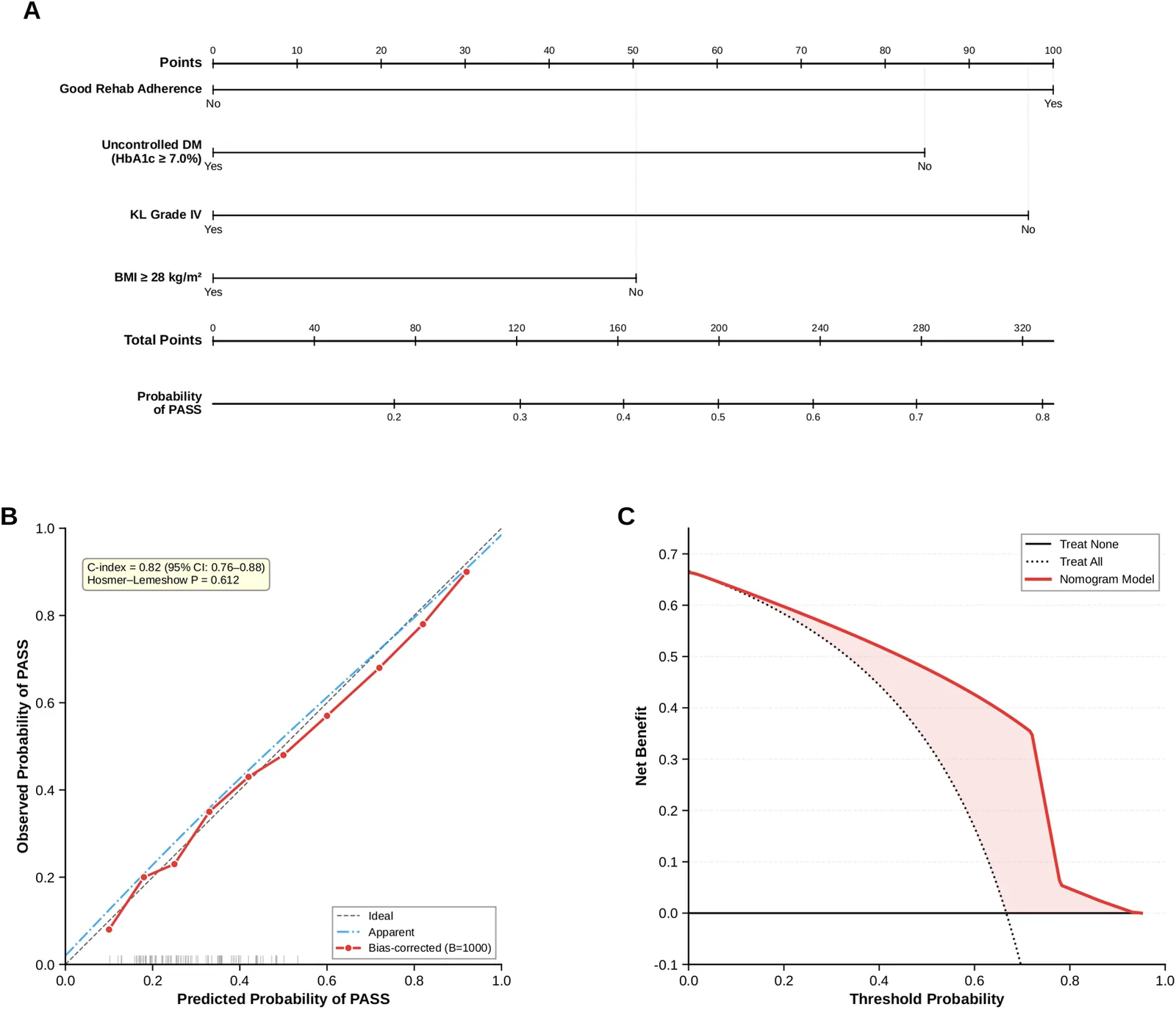
Development and internal validation of the nomogram for predicting PASS at 6 months. **(A)** Nomogram predicting the probability of achieving a Patient Acceptable Symptom State (PASS) at 6 months. Clinicians can locate the patient's status on each variable axis, draw a vertical line upward to the “Points” scale to get a score for each variable, and sum these scores to find the “Total Points.” A vertical line drawn downward from the “Total Points” axis provides the predicted probability of achieving PASS. **(B)** Calibration curve for the predictive model. The *x*-axis represents the predicted probability of achieving PASS calculated by the nomogram, and the *y*-axis represents the actual observed probability. The diagonal 45-degree dotted line represents perfect prediction. The red line (bias-corrected via 1000 bootstrap resamples) closely aligns with the ideal line, indicating excellent calibration. **(C)** Decision Curve Analysis (DCA) of the nomogram. The *y*-axis measures the net clinical benefit. The solid red line represents the predictive nomogram model. The dotted line assumes all patients will achieve PASS (Treat All), and the solid black horizontal line assumes no patients will achieve PASS (Treat None). The nomogram provides a higher net benefit across a broad range of threshold probabilities, demonstrating superior clinical utility. PASS, Patient Acceptable Symptom State; DM, diabetes mellitus; HbA1c, glycated hemoglobin; KL, Kellgren–Lawrence; BMI, body mass index; C-index, concordance index; CI, confidence interval; DCA, decision curve analysis.

## 4 Discussion

Using a 1:2 PSM design, the present study systematically evaluated the longitudinal clinical efficacy of intra-articular PN injection combined with prescribed rehabilitation therapy in patients with KOA with and without T2DM after effective control of confounding factors such as age, BMI, and baseline disease severity. The results showed that, over 6 months of follow-up, no statistical difference was observed between the DM and non-DM groups in the improvement trajectories of VAS and WOMAC scores. Likewise, the two groups showed a high degree of consistency in MCID achievement rate, OMERACT-OARSI response rate, and PASS achievement rate. Further subgroup analysis revealed a moderating effect of baseline HbA1c level on early efficacy in patients with DM, whereas the predictive nomogram constructed using a two-stage LASSO-Logistic modeling strategy provided a visualized tool for individualized clinical decision-making. In addition, component-level review of the rehabilitation records showed that the documented implementation rates of the four prescribed rehabilitation activities were comparable between the DM and non-DM groups, reducing concern that the observed between-group outcome comparison was substantially influenced by unequal rehabilitation exposure. PASS was deliberately selected as the binary endpoint for model development because the nomogram was intended to estimate whether an individual patient would reach an acceptable absolute pain state at the 6-month endpoint. This differs conceptually from MCID, which captures the magnitude of improvement relative to baseline, and from the OMERACT–OARSI responder criteria, which represent a composite treatment response. Accordingly, MCID and OMERACT–OARSI response were retained as complementary clinical outcomes rather than used as the primary modeling endpoint.

Our findings suggest that, when an adequate course of PN injection is administered and combined with standardized rehabilitation training, sufficient therapeutic responsiveness is still preserved in the local joints of patients with T2DM. This result is in line with the direction of several recent clinical studies on PN. In a randomized controlled trial (RCT) including 60 patients with KOA, Kim et al. (21) found that intra-articular PN injection was comparable to high-molecular-weight HA in relieving weight-bearing pain and improving WOMAC score, with favorable safety. In an earlier RCT, Vanelli et al. (22) likewise confirmed in 60 patients with KOA that PN injection was comparable to HA in pain relief, thereby providing the first randomized controlled evidence for the clinical use of PN. In a multicenter three-arm RCT, Moon et al. (23) further found that PN was even superior to conventional HA and cross-linked HA in alleviating weight-bearing pain. In a systematic review and meta-analysis including 12 studies, Elphingstone et al. (12) reported that PN was superior or non-inferior to HA with respect to pain relief and functional improvement. A large multicenter real-world study conducted in Korea across 17 orthopedic centers also confirmed that PN injection can provide significant and sustained pain relief for up to 6 months in routine clinical practice, and that no obvious attenuation of efficacy was observed in subgroups with metabolic comorbidities (11). In addition, a double-blind RCT by Dallari et al. (24) showed that PN combined with HA was significantly superior to HA alone in reducing pro-inflammatory cytokine levels in synovial fluid and improving joint function, suggesting that PN possesses independent biological therapeutic value. The present findings further extend these observations and, under the more rigorous design afforded by PSM control of confounders, provide the first validation of the comparable efficacy of PN combined with rehabilitation in patients with T2DM-associated KOA.

It is noteworthy that previous studies have not reached a consistent conclusion as to whether T2DM affects the efficacy of conservative treatment for KOA. Some studies have suggested that patients with diabetes respond less favorably to HA injection, which may be related to aggravated synovial inflammation and accelerated HA degradation in a hyperglycemic environment (7). However, the A2A receptor-mediated anti-inflammatory mechanism of PN does not depend on autologous blood quality or the biological activity of blood-derived products, which may constitute an important reason why its efficacy can still be maintained in patients with metabolic dysregulation (13, 25). A more in-depth examination at the molecular level suggests that the ability of PN to maintain efficacy in patients with T2DM-associated KOA may arise from its multilevel biological actions and their precise counteraction against the core pathological processes of diabetic arthropathy. Animal experiments have demonstrated that polydeoxyribonucleotide (PDRN) can significantly reduce the production of pro-inflammatory cytokines such as TNF-α and IL-6 in collagen-induced arthritis models through activation of the A2A receptor, thereby alleviating the severity of articular cartilage erosion (26). In lymphocytes from patients with arthritis, activation of A2A and A3 adenosine receptors has been shown to effectively inhibit the NF-κB signaling pathway and significantly reduce the release of matrix metalloproteinases such as MMP-1 and MMP-3 (27). More importantly, basic research has shown that adenosine A2A receptor signaling is a core regulatory mechanism for maintaining cartilage homeostasis. The chondrocyte transcriptomic profile of A2A receptor knockout mice closely resembles that of human osteoarthritic chondrocytes, characterized by upregulation of pro-inflammatory genes, downregulation of matrix organization genes, and aggravated oxidative stress, whereas exogenous A2A receptor agonists can effectively reverse these pathological changes (28). PN/PDRN has also been demonstrated *in vitro* to directly protect the cartilage matrix from degradation by slowing matrix metalloproteinase activity (29). In addition, all patients in the present study received a standardized rehabilitation program, and the fundamental supportive role of rehabilitation intervention in maintaining treatment efficacy should not be overlooked. On the other hand, stable HbA1c levels were observed in the DM group before and after intervention (baseline: 6.95 ± 0.85% vs. 6 months: 6.90 ± 0.90%), which is consistent with the findings of Habib et al. (30) regarding the impact of intra-articular injection on glycemic metabolism in patients with DM, thereby supporting the favorable endocrine safety profile of PN injection in patients with T2DM. Compared with the marked glycemic fluctuations caused by corticosteroid injection (10), this constitutes an important clinical advantage of PN in this special population.

Although the overall efficacy in the DM group was comparable to that in the non-DM group, the subgroup analysis of the present study revealed an important stratification phenomenon. Within the DM group, patients with baseline HbA1c < 7.0% exhibited significantly greater WOMAC improvement at 3 months and a significantly greater reduction in WOMAC from 0 to 3 months than patients with HbA1c ≥ 7.0%. At the 6-month endpoint, the gap between the two subgroups persisted in terms of PASS achievement rate (75.00% vs. 52.38%) and OMERACT-OARSI response rate (79.17% vs. 59.52%). This finding is consistent with basic research evidence regarding the pathological interaction between T2DM and KOA. Berenbaum and colleagues previously pointed out that osteoarthritis possesses features of a metabolic disease (3), and that AGEs can accumulate extensively in fibroblast-like synoviocytes under hyperglycemic conditions, significantly promoting inflammatory progression in KOA (5). The review by Veronese et al. systematically summarized multiple pathways through which T2DM accelerates cartilage degeneration, including AGEs accumulation, insulin resistance, and oxidative stress (7). In the pathological microenvironment of T2DM-associated KOA, persistent hyperglycemia can impair glucose transporter function in chondrocytes and induce mitochondrial oxidative stress, leading to excessive accumulation of ROS, which in turn drives cartilage matrix degradation and apoptosis (31). More recent research has further revealed that osteoarthritic chondrocytes exhibit marked dysregulation of energy metabolism, characterized by impaired mitochondrial respiration and abnormal enhancement of glycolysis, and these alterations are more pronounced in patients with concomitant metabolic syndrome (32). These pathological processes are likely to be more active in patients with HbA1c ≥ 7.0% and may partially offset the A2A receptor-mediated anti-inflammatory effect of PN, thereby leading to a slower rate of early response in this subgroup. It should be emphasized that, although the HbA1c ≥ 7.0% subgroup showed a slower early improvement rate and a lower endpoint achievement rate, clinically meaningful absolute improvement was still observed at 6 months (WOMAC decreased from 56.95 to 27.95). This suggests that PN combined with rehabilitation therapy is not ineffective in patients with poor glycemic control; rather, the upper limit of therapeutic benefit appears to be constrained by the metabolic burden. In an RCT involving 228 patients with T2DM-associated KOA, Su et al. (33) also found that exercise-based rehabilitation improved joint function while simultaneously reducing HbA1c, suggesting the possibility of bidirectional benefit between glycemic management and joint treatment. This concept is further supported by emerging evidence regarding metformin. A recent randomized, double-blind, placebo-controlled trial reported that 6 months of metformin treatment produced a modest but statistically significant reduction in knee pain and improvement in WOMAC outcomes in patients with symptomatic KOA and overweight or obesity (34). Although that trial was not specifically conducted in patients with T2DM, these findings raise the possibility that metformin may provide symptomatic benefits beyond its glucose-lowering role. In patients with T2DM-associated KOA, targeted metformin therapy could therefore potentially improve glycemic control while simultaneously providing an additional symptomatic benefit.

In the present study, 15 candidate variables were reduced by LASSO penalized regression, and four core predictors were identified to construct a nomogram for predicting the probability of achieving PASS. Among these, good rehabilitation adherence was the only independent protective factor (OR = 2.95), which is consistent with the evidence-based consensus in the field of KOA rehabilitation that systematic exercise rehabilitation constitutes the core of non-surgical management (17). Animal experiments have also demonstrated that active exercise can suppress ADAMTS-5 expression by inducing irisin release (35). At the clinical level, the observed association between good rehabilitation adherence and PASS achievement suggests that adherence to the prescribed rehabilitation program may contribute to favorable outcomes within the combined treatment pathway. However, because no PN-only comparator was included, the independent contribution of rehabilitation and any synergistic interaction between PN injection and rehabilitation could not be determined. Regarding the three independent risk factors, the predictive effect of poor glycemic control (HbA1c ≥ 7.0%, OR = 0.40) was consistent with the aforementioned subgroup analysis, again underscoring the importance of glycemic management. KL grade IV (OR = 0.35) suggests that the scope for biological therapy is fundamentally restricted when severe structural joint destruction is present, which is in line with previous findings on viscosupplementation (36); BMI ≥ 28 kg/m^2^ (OR = 0.58) reflects the unfavorable effect of excessive mechanical loading and adipose tissue-derived pro-inflammatory mediators on treatment efficacy (7). Previous studies have similarly shown that obesity and radiographic severity are core determinants predicting treatment failure (36), and that metabolic syndrome imposes additional pathological burden on joints through adipokine dysregulation and chronic low-grade inflammation (37). The A2A receptor-dependent anti-inflammatory and pro-reparative characteristics of PN may exert targeted intervention precisely at this level (13). From the perspective of clinical application, the present nomogram may assist in pre-treatment risk stratification. For patients with high BMI, KL grade IV, and HbA1c ≥ 7.0%, intensified metabolic management or evaluation for surgical indications may be preferentially recommended. By contrast, patients with KL grades II–III, good glycemic control, and high adherence may be more suitable candidates for this combined conservative strategy.

Nevertheless, several limitations of the present study should be acknowledged. First, as a retrospective study, although known confounding factors were controlled by PSM, the influence of unmeasured confounders cannot be completely excluded. Second, outcome measures were mainly based on subjective clinical scores (VAS and WOMAC), and objective assessment data such as synovial fluid biomarkers (e.g., AGEs, MMP-13, and type II collagen degradation fragments) and quantitative imaging findings (e.g., magnetic resonance imaging T1*ρ*/T2 mapping) were lacking (38). Consequently, the tissue-modifying effect of PN could not be verified at the microscopic level. In addition, the 6-month follow-up period was insufficient to evaluate the long-term durability and potential disease-modifying effect of this combined treatment; future prospective studies with longer follow-up are therefore needed. Finally, rehabilitation adherence was assessed on the basis of medical records and patient self-report, and some degree of recall bias may therefore have been introduced. Future studies may consider the use of wearable devices for objective monitoring. The present investigation was a single-center study with a relatively limited sample size, and the generalizability of the conclusions remains to be confirmed by multicenter studies with larger samples. In addition, the present study did not include a comparator group receiving PN injection without prescribed rehabilitation. Therefore, although favorable outcomes were observed among patients treated with the combined regimen, the independent effects of PN injection and rehabilitation, as well as any potential synergistic interaction between the two components, could not be separately quantified. Patients who were not referred for rehabilitation may also have differed systematically from those included in the present cohort, and incorporating such patients retrospectively without adequate baseline and follow-up comparability could introduce substantial selection bias. Future prospective controlled studies should include PN-only and combined-treatment groups to determine the incremental contribution of rehabilitation. Moreover, considering our finding that poorer glycemic control was associated with a lower probability of achieving PASS, a future prospective study specifically enrolling patients with T2DM-associated KOA would be valuable. Such a study could compare PN plus rehabilitation under standard diabetes management with the same combined orthopedic treatment accompanied by protocolized metformin-based glycemic management, preferably with stratification according to baseline HbA1c and BMI. We hypothesize that optimized glycemic control may improve the response to PN plus rehabilitation, while the potential anti-inflammatory and analgesic effects of metformin may provide an additional, although likely modest, improvement in pain and function. Serial assessment of HbA1c, VAS, WOMAC, PASS, and relevant metabolic or inflammatory biomarkers would help distinguish whether any additional benefit is mediated predominantly by improved glycemic control or by a direct disease-modifying or analgesic effect of metformin. However, this hypothesis requires prospective randomized validation and cannot be inferred from the present retrospective data.

In summary, through a propensity score-matched cohort design, the present study demonstrated that intra-articular PN injection combined with prescribed rehabilitation therapy can achieve clinical efficacy in patients with KOA complicated by T2DM comparable to that observed in non-DM patients, without adversely affecting glycemic metabolism. Subgroup analysis suggested that baseline HbA1c < 7.0% was associated with faster early response and a higher endpoint achievement rate. The predictive nomogram developed on the basis of the LASSO-Logistic strategy identified rehabilitation adherence, glycemic control status, radiographic grade, and BMI as the core predictors of PASS achievement, thereby providing a practical clinical tool for individualized therapeutic decision-making and prognostic evaluation in this patient population.

## Data Availability

The raw data supporting the conclusions of this article will be made available by the authors, without undue reservation.
