# Supplementary material for: Comparative efficacy of intra-articular polynucleotide injection combined with rehabilitation therapy in knee osteoarthritis with and without diabetes mellitus and construction of a nomogram: a propensity score-matched cohort study
Source: Front Med (Lausanne). 2026 Sep 25;13:1906042. doi: 10.3389/fmed.2026.1906042 (PMC13649387; doi:10.3389/fmed.2026.1906042)
Supplement: Supplementary file 1 [file Table1.docx]

**Supplementary Table S1. Candidate variables entered into the LASSO regression and their selection status**

| **Candidate variable** | **Coding/type** | **LASSO selection status** |
| --- | --- | --- |
| Age | Continuous, years | Coefficient shrunk to zero |
| Sex | Female vs. male | Coefficient shrunk to zero |
| BMI ≥ 28 kg/m² | Binary | Retained |
| Symptom duration | Continuous, years | Coefficient shrunk to zero |
| KL grade IV | Grade IV vs. grades II–III | Retained |
| Bilateral knee involvement | Binary | Coefficient shrunk to zero |
| T2DM status | Binary | Coefficient shrunk to zero |
| Uncontrolled DM | HbA1c ≥ 7.0% vs. non-DM/well-controlled DM | Retained |
| Hypertension | Binary | Coefficient shrunk to zero |
| Hyperlipidemia | Binary | Coefficient shrunk to zero |
| Baseline VAS | Continuous, 0–10 | Coefficient shrunk to zero |
| Baseline WOMAC | Continuous, 0–96 | Coefficient shrunk to zero |
| Number of intra-articular PN injections | Continuous, 3–5 | Coefficient shrunk to zero |
| Rehabilitation adherence | Good vs. poor | Retained |
| Concomitant oral NSAID use | Yes vs. no | Coefficient shrunk to zero |
